# Assessment of Household Food Insecurity During a Medical Mission to Chincha, Peru

**DOI:** 10.7759/cureus.17224

**Published:** 2021-08-16

**Authors:** Matthew K Edwards, Manuel Valdivieso, Julio A Leey, Jessica Portillo-Romero

**Affiliations:** 1 School of Medicine, Case Western Reserve University, Cleveland, USA; 2 Medicine, University of Michigan, Ann Arbor, USA; 3 Medicine, University of Florida, Gainesville, USA

**Keywords:** food insecurity, household food security survey, medical mission, underserved population, community health

## Abstract

Introduction

Food insecurity directly influences health outcomes and is an important consideration for medical missions seeking to address chronic disease, particularly those serving disaster-prone communities. The region of Peru in which we held an inaugural mission is vulnerable to developing food insecurity following natural disasters. We, therefore, sought to evaluate food insecurity to understand the community’s needs and inform future public health efforts.

Methods

In this cross-sectional pilot study, a convenience sample representing the households of patients attending a student-run health fair at the community medical center in Chincha, Peru was assessed for food insecurity. An adult female (*n* = 30) of each randomly selected family attending the fair was asked to complete the Household Food Security Survey (HFSS) developed by the US Department of Agriculture. The survey items were aggregated into a single, continuous food security scale reflecting the severity of hunger within a household.

Results

Two-thirds of respondents (*n* = 20) acknowledged anxiety about having enough food at home over the past 12 months, making it the most common concern. Nearly three in five respondents were concerned about their ability to provide a balanced diet. We found that 16.7% of all households were food insecure with severe hunger, 26.7% were food insecure with moderate hunger, 30% were food insecure without hunger, and 26.7% were food secure.

Conclusion

Nearly three-quarters of families attending our clinic experience some degree of food insecurity. Families with children were disproportionately affected. The high levels of food insecurity many years after a natural disaster support the development of future social programs such as food pantries. We intend to continue our partnership in Chincha and perform the HFSS survey on a periodic basis to monitor hunger.

## Introduction

In 2007, an 8.0 magnitude earthquake destroyed many lives and the medical infrastructure of Chincha, Peru, a city of approximately 56,000 individuals located 200 kilometers south of the capital, Lima. Following the earthquake, the Peruvian American Medical Society (PAMS) helped to build a primary care center in Chincha that would address the clinical needs of its population. This facility provides primary care services daily, some specialty care weekly, and hosts four to five medical mission trips a year. These missions are coordinated by numerous PAMS Chapters and members as well as several universities from the United States. The missions reinforce the local care provided and often offer new specialties that improve the treatment of chronic diseases such as gastrointestinal disorders, hypertension, and diabetes. However, one of the most enduring health challenges following a disaster is food insecurity, defined as the uncertain or restricted availability of food of sufficient nutritional quality, which can be acquired by socially acceptable means [1]. With the destruction of rural infrastructure and agriculture by an earthquake, pre-existing health disparities driven by socioeconomic status worsen [2-4].

As of 2015, 23% of the Peruvian population was deemed at high or very high risk of developing food insecurity in the event of subsequent natural disasters [5]. In Ica, the region in which the city of Chincha is found, nearly 1.4% of the population was highly or very highly food insecure as of 2015 [5]. Food insecurity is tightly associated with health outcomes in all age groups and should be assessed during medical missions seeking to lower the burden of chronic disease [6,7]. Measuring food insecurity, therefore, is critical in this community. A food insecurity questionnaire was integrated as a pilot study into a student-run medical mission to the Chincha medical center in 2020. 

## Materials and methods

This project was designed as a cross-sectional pilot study, sampling the households of patients attending our physician-supervised student-run health fair at the PAMS Polyclinic community medical center in Chincha, Peru in March 2020. More than 668 patients were screened in one week for diabetes, hypertension, anemia, and visual impairment, with an additional food insecurity survey performed within a convenience sample of these patients. An adult female representative (*n* = 30) of each randomly selected family was asked to complete a Spanish translation of the Household Food Security Survey (HFSS) developed by the US Department of Agriculture (USDA) [1]. Spanish-speaking facilitators were available to assist participants with literacy barriers. This questionnaire was chosen for our study because it includes specific questions that distinguish answers for adults (hunger and skipped meals) from children (reducing the size of the child’s meal, skipping the child’s meal, the child not eating enough, the child being hungry, and the child not eating for one day). The HFSS typically requires less than 4 minutes to complete and consists of 18 questions for households with children and 10 questions for households without children [1]. The survey items can be aggregated into a single, continuous food security scale ranging from 0 to 10, reflecting the severity of hunger within a household. The food security scale can be further simplified into four categories: Food Secure, Food Insecure without Hunger, Food Insecure with Hunger - Moderate, and Food Insecure with Hunger - Severe [1].

Food-secure households demonstrate little to no evidence of food insecurity. Households that are food insecure without hunger exhibit concern for inadequate food supply that drives changes like the reduction of food quality, yet there is no significant reduction in food intake. Moderate food insecurity with hunger entails reduced food consumption by adults that causes recurrent hunger, yet does not directly affect the children. Households with severe food insecurity see reduced food intake by the children and extreme reductions for the adults.

Affirmative and negative survey responses were coded to the values of “1” and “0,” respectively, and the questions were ordered from least to most severe. Surveys with missing values underwent a methodologically conservative and validated process of imputation in which false positives were minimized [1]. Missing values were replaced with “1” if there was at least one affirmative response to a question more severe than the imputed item and no negative responses to items less severe. All other missing values were replaced with “0.” This method assumes that households that affirm a question will affirm all less severe questions, and those that deny a question will deny all more severe questions [1].

Households filled out the questionnaire anonymously and no patient identifiers were collected. All analyses were conducted using R statistical software. Two-sided *t*-tests were conducted to determine differences in mean food security scale value between households with and without children.

## Results

Of the 30 female patients evaluated in our study, 19 (63%) had one or more children (younger than 18 years) in their household and were thus administered the full 18-item survey, while the remaining 11 (37%) were given the shortened 10-item survey for adult-only households. Surveys were prematurely discontinued in 7 (23%) of the respondents, therefore requiring data imputation as detailed in the Methods section.

Table 1 provides percentages of respondents that affirmed each item of the survey. Items 1 and 2 regarded anxiety for insufficient food supply; items 3, 4, and 5 pertained to perception of inadequate food quality; items 6-8, 10, 11, 13, and 15 were related to adult food intake and consequences; and items 9, 12, 14, and 16-18 reported child food intake and consequences. Two-thirds of respondents acknowledged anxiety about having enough food at home over the past 12 months, making it the most common concern. Nearly three in five respondents were concerned about their ability to provide a balanced diet. Across the food intake items, similar proportions of respondents reported reductions for adults (34.8%) and children (27.2%) in the household, and there was no significant difference between these grouped items (p > 0.05). However, proportions were skewed toward lower-severity food insecurity items.

**Table 1:** Distribution of questions on the Food Security Scale, ordered in increasing severity, among households in Chincha, Peru, 2020.

	Items	Question Description	Affirmative Responses
Food Supply Anxiety	1	Worried that food would run out before you are able to afford more	20/30 (67%)
	2	Purchased food would not last until resupply	20/30 (67%)
Quality Inadequacy Perception	3	Few kinds of low-cost food were available	11/19 (58%)
	4	Could not afford to eat balanced meals	21/30 (70%)
	5	Could not feed children a balanced meal	10/19 (53%)
Reduced Adult Food Intake	6	Adults reduced or skipped meals	13/30 (43%)
	7	You ate less than felt you should	17/30 (57%)
	8	Adults frequently reduced or skipped meals	5/30 (17%)
	10	You were hungry but did not eat	15/30 (50%)
	11	You lost weight because of not enough food	10/30 (33%)
	13	Adults would not eat for whole day	9/30 (30%)
	15	Adults frequently would not eat for whole day	4/30 (13%)
Reduced Child Food Intake	9	Children were not eating enough	12/19 (63%)
	12	Reduced the size of children’s meals	6/19 (32%)
	14	Children have ever been hungry	6/19 (32%)
	16	Children have ever skipped meals	4/19 (21%)
	17	Children frequently skipped meals	2/19 (11%)
	18	Children did not eat for a whole day	1/19 (5%)

**Figure 1:**
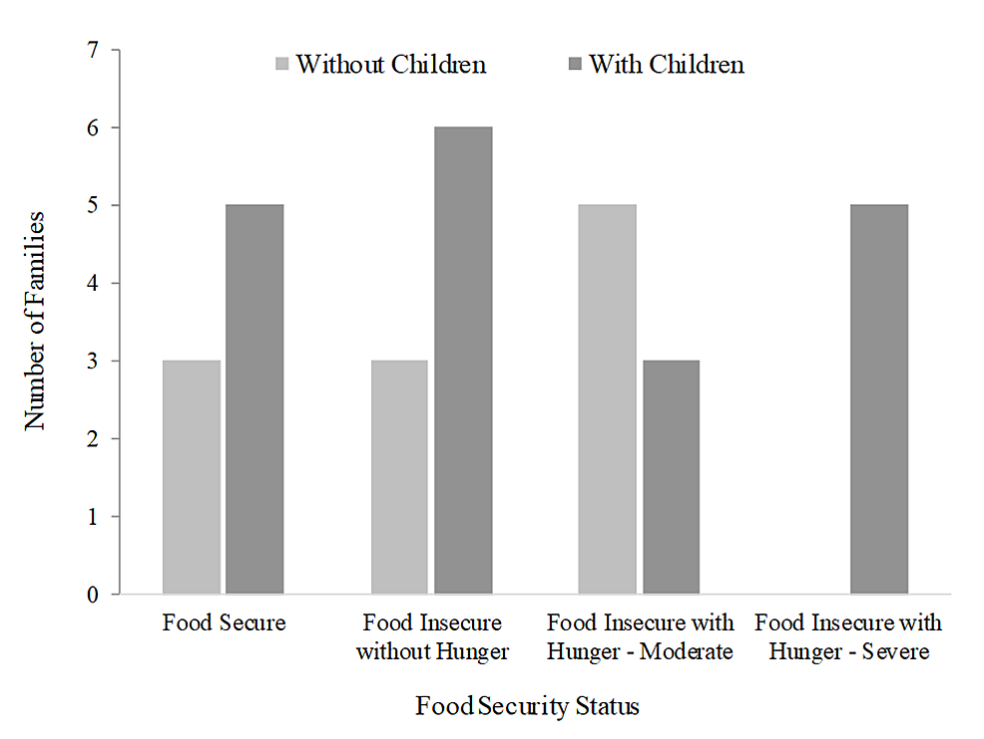
Distribution of households without children and with children representing each category of food security status.

As depicted in Figure 1, 16.7% of all households were found to be food insecure with severe hunger, 26.7% were food insecure with moderate hunger, 30% were food insecure without hunger, and 26.7% were food secure. There were no instances of food insecurity with severe hunger in adult-only households. Of households without children, 45.5% experienced food insecurity with moderate hunger, 27.3% had food insecurity without hunger, and 27.3% were food secure. For households with children, 26.3% were food insecure with severe hunger, 15.8% were food insecure with moderate hunger, 31.6% were food insecure without hunger, and 26.3% were food secure. The average food security scale value across all households was 4.0 (SD 0.46) and was higher in households with children (4.1) than households without children (3.7), though this difference was not significant (p > 0.05).

## Discussion

Chronic food deprivation afflicts more than 820 million people globally, many of whom live in the developing nations most susceptible to the rising cost of food and the disruption of food supplies by natural disasters [8-10]. The Peruvian Ministry of Development and Social Inclusion (MIDIS) estimates that 31% of Peru’s population experiences medium to very high levels of food insecurity [5]. The city of Chincha lies within the administrative department of Ica, which faces considerable risk of food insecurity due to the occurrence of natural phenomena [11]. The findings of this study corroborate these characterizations, with nearly three-quarters of our sampled families experiencing some degree of food insecurity. This high level of food insecurity is consistent with a study by Pillaca-Medina, which used the USDA’s HFSS in a probabilistic sample from nearby towns. They found food insecurity in 53-66% of the populations and attributed their results to low incomes, inadequate education, fluctuations in food prices, and natural disasters including earthquakes [11]. In contrast to other reports [5,11], ours focused on patients seeking medical care in a community center, and thus is not comparable to prevalence studies in the general population.

This study also found that families with children tended to have higher values of food insecurity than adult-only households. These results align with previous studies, including surveys of US households performed by the USDA’s Economic Research Service in 2016, which revealed that 16.5% of households with children experienced food insecurity, compared to the national average of 12.3% [12]. Food insecurity in children is particularly worrisome because it is associated with poor diet, impaired cognitive function, and the development of chronic disease in adulthood [13]. Despite the increased likelihood of food scarcity in households with children, studies have consistently observed the behavior of adults undergoing relatively severe levels of hunger before hunger first appears in children [1]. This pattern was generally borne out in our study, with the exception of a single case in which the respondent denied eating less than they felt they should or being hungry, yet reported that their children had skipped meals and had been hungry because the household could not afford more food.

Studies using the HFSS in the United States have shown that approximately 0.5-1% of respondents will answer “don’t know” or refuse to answer any question of the survey [1]. Our survey experienced a relatively high proportion of non-responses, with 23% of surveys being discontinued. We attribute these findings to the emotional distress exhibited by some of the respondents as they were filling out their questionnaires. The discrepancy between our experience and the non-response rate from other reports may be due to cultural differences or sudden self-awareness of food insecurity while seeking medical care. Whether or not this is random or causal is unknown and deserves further investigation. 

The USDA’s HFSS is a powerful tool that accounts for the complex, multidimensional character of food insecurity, while also providing a measure that is feasible for use in locally designed surveys [1]. The core module of the food security survey has been used to successfully monitor hunger throughout North America for decades, and has further been applied by studies in such disparate locations as Ethiopia, India, Peru, and Israel [8,9,11,14]. Our study is limited by its use of the Spanish version of the HFSS, however, due to a lack of local cultural adaptation of the tool. Vargas and Penny performed a mixed-method study that allowed them to adapt the HFSS to communities throughout Peru [15]. By rewording survey questions and identifying site-specific vulnerabilities, this altered version of the HFSS likely allowed the authors to more accurately assess perceptions, attitudes, and experiences of food insecurity [15].

Other limitations of our study include its low sample size and single-center selection of patients, which may limit the finding’s external validity. Additionally, the HFSS was not designed to determine the nutritional adequacy of the household diet. Although it is likely that households with high food insecurity values will also have less balanced meals, the importance of a diet’s nutritional content makes additional study of this dimension imperative [1].

Despite this study’s limitations, it has great value in guiding future actions that address food insecurity in underserved communities in the aftermath of natural disasters. The presence of high levels of food insecurity several years after an earthquake supports the development of social programs such as food pantries. The fact that nearly three-quarters of this patient population experiences food insecurity of various degrees could predict poor compliance, especially in settings where patients have to share the cost of medical care.

## Conclusions

When the HFSS is used on a periodic basis, it serves as a tool to monitor hunger and the progress of programs that combat food insecurity. Consistent with the MIDIS recommendations to investigate factors affecting food insecurity and disseminate information on vulnerable areas, our program intends to continue its partnership in Chincha to understand and eventually address its causes of hunger. Given that food insecurity was common among patients seeking medical attention at this community care center, the impact of food security on treatment adherence and medical care warrants further evaluation.
